# Successful Confinement of a Familial Cluster of COVID-19 in Qingdao, China, in the Early Phase of Pandemic

**DOI:** 10.1017/dmp.2020.489

**Published:** 2020-12-22

**Authors:** Jing Jia, Jiwei Liang, Xueling Xin, Xiaoqi Dai, Bi Hao, Hualei Xin, Xuekui Li, Yalin Han, Bei Pan, Xiaowen Hu, Fachun Jiang, Ruqin Gao, Huaqiang Zhang

**Affiliations:** Municipal Centre of Disease Control and Prevention of Qingdao, Qingdao Institute of Prevention Medicine, Qingdao City, Shandong Province, People’s Republic of China

**Keywords:** COVID-19, outbreak, familial cluster

From January 26, 2020 to February 3, 2020, a total of 7 confirmed cases and 2 asymptomatic infected persons were reported in Qingdao, China. These cases had no history of sojourning in Hubei Province, contact with animals, visits to markets, or eating game meat.

On January 19, 2020, patient 1, the initial case, returned to Qingdao from Kunming by air, a flight on which there were 2 persons from Wuhan City with fever. She developed fever, cough, and weakness on January 21, 2020. She went to an outpatient clinic near her community and was treated with intravenous cefazolin from January 23 to 24. Due to persistent symptoms, she was admitted to hospital on January 25, 2020, and diagnosed as a suspected case. The throat swab tested positive for coronavirus disease 2019 (COVID-19) by reverse transcriptase polymerase chain reaction (RT-PCR) on January 26, 2020.

**Figure 1.**
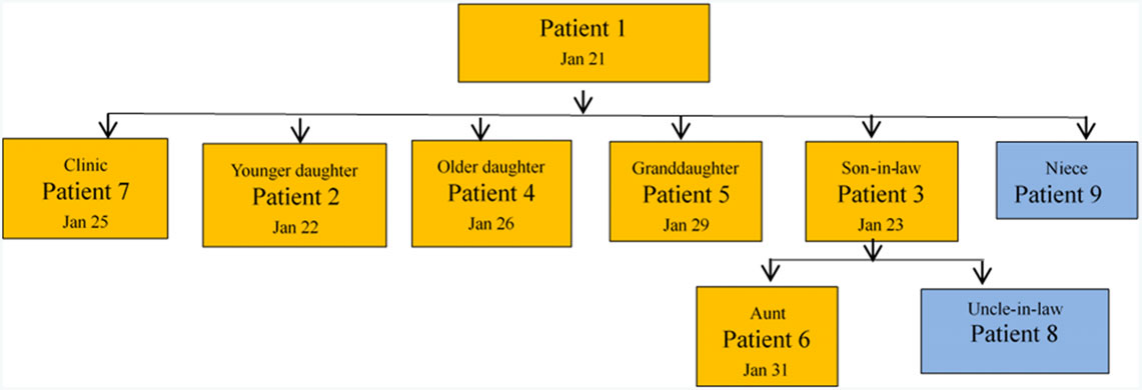
The relation diagram of cases in the cluster. Textboxes filled in orange are confirmed cases, blue are asymptomatic infected persons; the date in each textbox is the date of onset.

**Figure 2.**
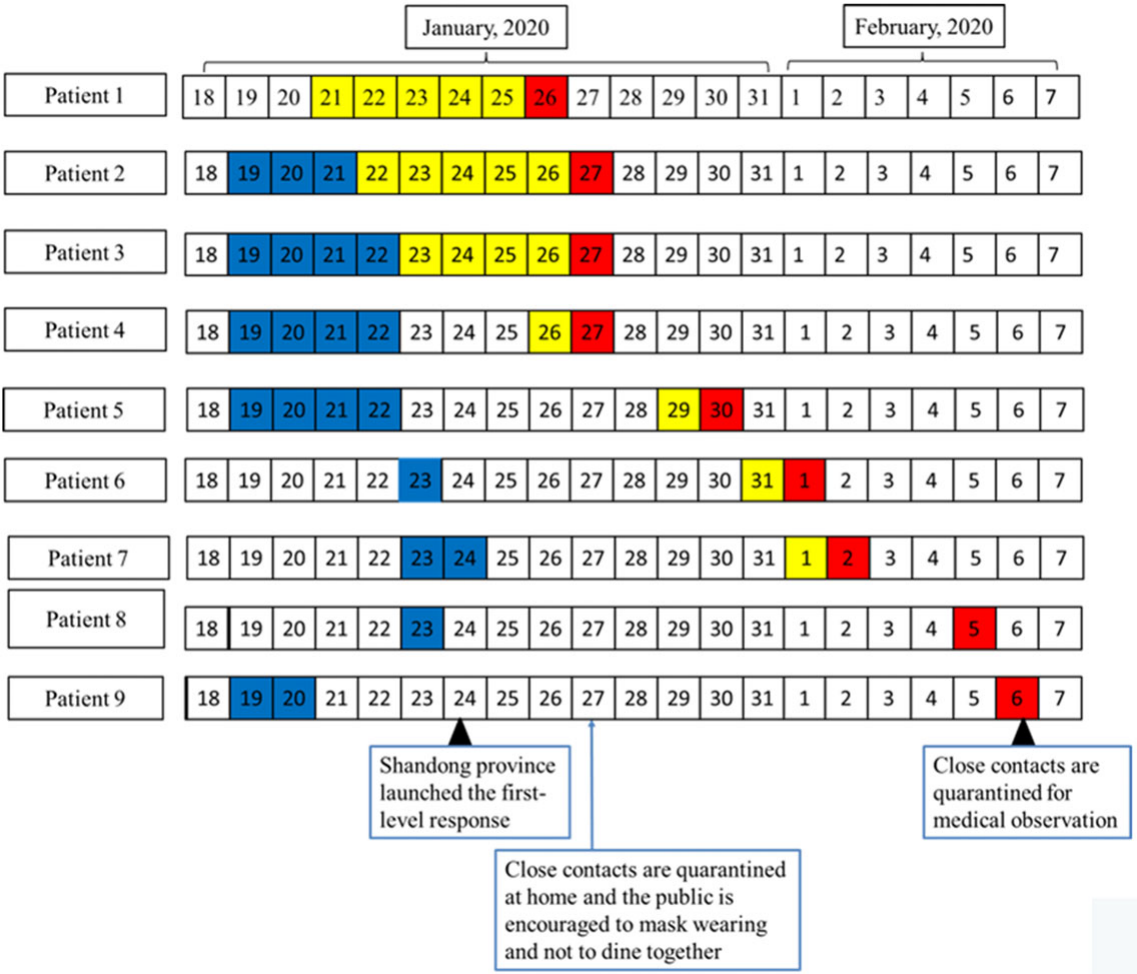
The sequence chart of onset of the cluster and their contacts in January 2020. Dates filled in red are confirmed times, in yellow are onset times, in blue are exposure times to patient 1, and green are exposure times to patient 3.

Patient 2 lived together with her mother (patient 1), and the older daughter of patient 1 (patient 4) lived in the same community. Patient 1 had gone to the house of patient 4 many times since she returned to Qingdao. On January 20, patient 1 had lunch in her father’ house, which was in another community, with her father, sister, niece (asymptomatic infected person, patient 9), and granddaughter (patient 5). In the evening, the 2 daughters (patients 4 and 2), son-in-law (patient 3, husband of patient 4), grandson, and granddaughter (patient 5) had supper with patient 1 in the house of patient 4. Patient 3 and 4 took their children back to their parents’ home for the Spring Festival on January 23. The family visited their aunt (patient 6) and uncle-in-law (asymptomatic infected person, patient 8) on the evening of January 23. None wore surgical masks during the entire visit. Patient 7, who also did not wear a surgical mask, met patient 1 in the same clinic when patient 1 attended to the outpatient clinic (Figures 1 and 2). Due to the limited conditions in the early stage of the epidemic, nucleic acid testing lagged behind. If the nucleic acid testing of Case 9 was carried out before February 6, it would be more conducive to the prevention and control of the epidemic.

Through the field investigation, 115 persons were exposed during this cluster event, and the total infection rate was 6.09% (7/115). The infection rate of only this family was 41.18% (7/17). All the exposed persons were sent to designated hospitals, and they were subjected to intensive isolation and medical observation since patient 1 was confirmed. After a maximum incubation period had passed, no more cases related to this cluster were reported, and the transmission of this cluster was terminated on February 3, 2020.

As the COVID-19 outbreak occurred, Shandong province launched the first-level response to this public health emergency, in accordance with the management as A class infectious disease, belonged to B class infectious disease.^1^ Meanwhile, Qingdao immediately entered a state of “war.” Since the first confirmed case of COVID-19 was reported, the number of cases had been increasing in Qingdao City. So far, 171 cases had been reported in Qingdao City, which included 118 confirmed cases and 53 asymptomatic infected persons. This cluster, as the first cluster in Qingdao, was of great significance for prevention and control of the epidemic. On the 1 hand, it further confirmed the importance of “4 early” (early detection, early identification, early isolation, and early treatment). The timely detection and isolation of cases and their close contacts could effectively prevent further spread of the epidemic, which was the key point of successful control of this cluster.^2^ On the other hand, this cluster raised people’s awareness of effective measures to prevent and control the epidemic, that is, they should not take it lightly and especially wear masks consciously and avoid eating meals together.^3^ Above all, Qingdao successfully controlled several clusters of outbreak, and no community transmission occurred. As shown in our study, it was crucial to detect and isolate cases and trace and quarantine close contacts as early as possible, which was particularly important when PCR results were delayed.

Our study had limitations. First, we only tested samples from patients. Samples from the environment were not obtained. Second, the isolated virus was not sequenced, and the homology analysis was not performed.
